# Hyaluronan expression in gastric cancer cells is associated with local and nodal spread and reduced survival rate

**DOI:** 10.1038/sj.bjc.6690180

**Published:** 1999-03

**Authors:** L P Setälä, M I Tammi, R H Tammi, M J Eskelinen, P K Lipponen, U M Ågren, J Parkkinen, E M Alhava, V-M Kosma

**Affiliations:** Department of Surgery, Kuopio University Hospital, Kuopio, Finland; Department of Anatomy, University of Kuopio, Kuopio, Finland; Departments of Pathology and Forensic Medicine, University of Kuopio, Kuopio, Finland; Departments of Pathology and Forensic Medicine, Kuopio University Hospital, Kuopio, Finland

**Keywords:** hyaluronan, gastric cancer, prognosis

## Abstract

Hyaluronan (HA), an extracellular high-molecular-mass polysaccharide, is supposed to be involved in the growth and progression of malignant tumours. We studied the cellular expression of HA in 215 operated stage I–IV gastric cancer patients using a specific biotinylated probe. Most (93%) of the tumours showed HA staining in their parenchyma, whereas the stroma inside and around the tumour was stained in every case. When HA expression was compared with the clinical and histological features of the tumours, a strong staining intensity in the tumour parenchyma was found to be associated with deep tumour invasion (pT3 or 4) and with mixed type of Laurén. A high proportion of HA-positive cells of all neoplastic cells was significantly associated with deep tumour invasion, nodal metastasis, positive lymphatic invasion, poor differentiation grade, as well as with inferior prognosis in univariate survival analysis. However, in multivariate analysis, only pT, pN, and vascular and lymphatic invasion emerged as independent predictors of survival in gastric cancer. The results indicate that ectopic HA expression is a frequent feature of gastric adenocarcinoma, and is associated with tumour progression and poor survival rate. © 1999 Cancer Research Campaign


*British Journal of Cancer* (1999) **79**(7/8), 1133–1138
© 1999 Cancer Research Campaign
Article no. bjoc.1998.0180

### Hyaluronan expression in gastric cancer cells is associated with local and nodal spread and reduced survival rate

**LP Setälä[1], MI Tammi[2], RH Tammi[2], MJ Eskelinen[1], PK Lipponen[3], UM Ågren[2], J Parkkinen[3,4], EM Alhava[1] and V-M Kosma[3,4]**

[1]Department of Surgery, Kuopio University Hospital; [2]Department of Anatomy, University of Kuopio; [3]Departments of Pathology and Forensic Medicine, [3]University of Kuopio and [4]Kuopio University Hospital

**Summary** Hyaluronan (HA), an extracellular high-molecular-mass polysaccharide, is supposed to be involved in the growth and progression of malignant tumours. We studied the cellular expression of HA in 215 operated stage I–IV gastric cancer patients using a specific biotinylated probe. Most (93%) of the tumours showed HA staining in their parenchyma, whereas the stroma inside and around the tumour was stained in every case. When HA expression was compared with the clinical and histological features of the tumours, a strong staining intensity in the tumour parenchyma was found to be associated with deep tumour invasion (pT3 or 4) and with mixed type of Laurén. A high proportion of HA-positive cells of all neoplastic cells was significantly associated with deep tumour invasion, nodal metastasis, positive lymphatic invasion, poor differentiation grade, as well as with inferior prognosis in univariate survival analysis. However, in multivariate analysis, only pT, pN, and vascular and lymphatic invasion emerged as independent predictors of survival in gastric cancer. The results indicate that ectopic HA expression is a frequent feature of gastric adenocarcinoma, and is associated with tumour progression and poor survival rate.

**Keywords:** hyaluronan; gastric cancer; prognosis

Hyaluronan (hyaluronic acid, HA), a high-molecular-mass glycosaminoglycan, is an abundant component of the extracellular matrix and pericellular coat. In embryonic tissue, it is believed to contribute to the primordial, loose extracellular matrix, to enhance cell migration and to modulate differentation (Pauli and Knudson, 1988; Toole, 1991; Turley, 1992). In neoplastic tissues, it presents with several functions as a moderator of tumour cell adhesion, migration and angiogenesis (Catterall et al, 1995; Rooney et al, 1995). Moreover, it has been suspected as functioning as a signal that stimulates transformation, tumour growth and metastasis (Pauli and Knudson, 1988; Catterall et al, 1995; Hall et al, 1995). HA has at least two important receptors on the cell surface, CD44 and RHAMM (Rudzki and Jothy, 1997). The interaction between CD44 and its main ligand HA is presumed to be associated with HA degradation (Underhill, 1992) and invasive potential (Rudzki and Jothy, 1997).

The stroma of most malignant tumours, including gastric cancer (Sowa et al, 1989; Noguchi et al, 1993; Wang et al, 1996), contains more HA than the corresponding normal tissue, although less is known of the expression of HA in the cancer cells (Knudson, 1996). However, the methods used in some of the earlier studies are less specific and cannot distinguish between HA derived from tumour parenchyma and that from the surrounding stroma (Knudson, 1996). Some gastric cancer cell lines are capable of secreting HA in vitro (Sobue et al, 1983), whereas histological analysis with a specific probe on a small number of cases suggested low expression of HA in gastric carcinoma cells (Wang et al, 1996).

As far as the authors are aware, there are no studies concerning the relation between HA expression and tumour spread or survival in gastric cancer, whereas several studies suggest that the HA receptor CD44 is associated with invasion and prognosis (Hong et al, 1995; Streit et al, 1996; Müller et al, 1997). The aim of the present study was to assess HA expression in gastric adenocarcinomas in a large cohort, consisting of 215 patients, and to study the association between HA expression and clinical and histopathological characteristics of the tumours. In addition, the prognostic significance of HA expression was evaluated in a long-term follow-up.

### PATIENTS AND METHODS

### Patients

The study was based on 215 patients diagnosed and treated for primary gastric cancer at Kuopio University Hospital between 1976 and 1988, and followed up until 1993. The clinical data were obtained from the patient records. The location and size of the tumour as well as the status of the regional lymph nodes and other intra-abdominal organs were registered as described on gastroscopy, at operation and in examination of the resected stomach (UICC, 1987). All patients were operated on, a total gastrectomy was performed in 96 cases (45%), a subtotal gastrectomy in 77 cases (36%) and a gastric resection in 41 cases (19%). One patient underwent only an explorative laparotomy. Additional treatment, such as radiation therapy or chemotherapy was given to 11 patients. The causes of death were obtained from the patient

*Received 24 February 1998*
*Revised 27 May 1998*
*Accepted 3 June 1998*

*Correspondence to:* L Setälä, Department of Surgery, Kuopio University Hospital, PL 1777, FIN-70211 Kuopio, Finland records and from the files of the Finnish Cancer Registry and General Statistical Office in Finland.

### Histological methods

The tumour samples were routinely fixed in 10% buffered formalin and embedded in paraffin. Several original sections from each of the primary tumours were re-examined and the most representative tissue block was selected, cut at 5-µm thickness and stained in haematoxylin and eosin (HE). The tumours were classified according to Laurén as diffuse, intestinal, mixed or unclassified (Laurén, 1965). All tumours except those of the diffuse type were graded as well differentiated (grade I), moderately differentiated (grade II) or poorly differentiated (grade III). The depth of tumour invasion (UICC, 1987) and invasion into the walls of veins, arteries and lymphatics, or into the perineural space was registered in all samples which contained the full thickness of gastric wall and graded as weak (+), moderate (++) or strong (+++) (Setälä et al, 1996). The infiltration of lymphocytes and plasma cells (TIL, tissue infiltrating lymphocytes) was estimated, avoiding ulcerated or necrotic areas, and graded as weak, moderate or strong.

### Preparation of the biotinylated HA probe

The biotinylated hyaluronan binding complex (bHABC) was prepared from bovine articular cartilage as described previously (Wang et al, 1992; Tammi et al, 1994*a*). This probe contains the biotinylated HA-binding G1 fragment of aggrecan and link protein, which stabilizes the ternary complex with HA. Polyacrylamide gel electrophoresis of the probe showed only bands corresponding to the HA-binding region of aggrecan and link protein. The 5-µm-thick sections of the samples were deparaffinized in xylene and rehydrated in graded alcohols, followed by washing in 0.1 M sodium phosphate buffer (PB), pH 7.4. The endogenous peroxidase activity was blocked by washing the slides with 0.3% hydrogen peroxide for 3 min and the non-specific binding by incubation with 1% bovine serum albumin (BSA) in PB for 30 min. The sections were then incubated overnight with the bHABC (5 µg $ml^{-1}$, diluted in 1% BSA) at 4°C, washed with PB and treated with avidin–biotin–peroxidase (Vector, Irvine, CA, USA; 1:200 dilution) for 1 h at room temperature. After washes in PB, the slides were incubated for 5 min in 0.05% 3,3′-diaminobenzidine (Sigma, St Louis, MC, USA) and 0.03% hydrogen peroxide in PB at room temperature. After the washes, the sections were counterstained and mounted in Depex.

Staining specificity was controlled by predigesting the sections with *Streptomyces* hyaluronidase (Seikagagu, Tokyo, 100 TRU $ml^{-1}$ in 0.1 M sodium acetate buffer, pH 5.0, for 3 h) in the presence of protease inhibitors (Tammi et al, 1994*b*) and preincubating the bHABC probe with hyaluronan oligosaccharides (Ripellino et al, 1985). No staining was observed in hyaluronidase or oligosaccharide control sections (Figure 1E).

**Table 1** Clinicopathological data of the 215 gastric cancer patients

| | |
|---|---|
| Depth of invasion | |
| pT1 | 32 (15%) |
| pT2 | 37 (17%) |
| pT3 | 126 (59%) |
| pT4 | 19 (9%) |
| pTX | 1 |
| Size of the tumour | |
| < 2 cm | 22 (10%) |
| 2–5 cm | 48 (22%) |
| 5–10 cm | 59 (27%) |
| > 10 cm | 41 (19%) |
| Not known | 45 (21%) |
| Laurén classification | |
| Diffuse | 101 (47%) |
| Intestinal | 85 (40%) |
| Mixed | 20 (9%) |
| Unclassified | 9 (4%) |
| Vascular invasion | |
| Negative | 195 (91%) |
| Positive | 17 (8%) |
| Not assessed | 3 (1%) |
| Perineural invasion | |
| Negative | 117 (54%) |
| Weak | 71 (33%) |
| Strong | 25 (12%) |
| Not assessed | 2 (1%) |
| Nodal status | |
| pN0 | 96 (45%) |
| pN1 | 88 (41%) |
| pN2 | 16 (7%) |
| pNX | 15 (7%) |
| Location of the tumour | |
| Proximal third | 34 (16%) |
| Middle third | 31 (14%) |
| Distal third | 111 (52%) |
| More than one-third | 38 (18%) |
| Not known | 1 |
| Differentiation grade | |
| Grade I | 28 (13%) |
| Grade II | 54 (25%) |
| Grade III | 29 (13%) |
| Not assessed | 104 (48%) |
| TIL | |
| Weak | 126 (59%) |
| Moderate | 64 (30%) |
| Strong | 16 (7%) |
| Not assessed | 9 (4%) |
| Lymphatic invasion | |
| Negative | 117 (54%) |
| Weak | 61 (28%) |
| Strong | 35 (16%) |
| Not assessed | 2 (1%) |
| Metastasis | |
| M0 | 181 (84%) |
| M1 | 26 (12%) |
| MX | 8 (4%) |

TIL, tumour-infiltrating lymphocytes and plasma cells.

### Assessment of HA staining

All samples were analysed by a pathologist (VMK) without knowledge of other clinical or histological data. The staining intensity in the tumour parenchyma and in the stroma around and inside the malignant areas was registered as absent (–), weak (+), moderate (++) or strong (+++) from the most representative tissue section, using the ‘normal’ weak staining of the stroma as an internal positive control (Figure 1). The percentage of HA-positive tumour cells of all neoplastic cells in the section was also

*© Cancer Research Campaign 1999*

**Table 2** Hyaluronan expression in 215 gastric cancer patients

| Staining | No. of cases | % |
|---|---|---|
| Stroma | | |
| Negative | 0 | 0 |
| Weakly positive | 7 | 3 |
| Moderately positive | 42 | 20 |
| Strongly positive | 166 | 77 |
| Tumour parenchyma | | |
| Negative | 15 | 7 |
| Weakly positive | 30 | 14 |
| Moderately positive | 99 | 46 |
| Strongly positive | 71 | 33 |
| The percentage of HA-positive cells | | |
| 0 | 15 | 7 |
| < 30 | 105 | 49 |
| 30–100 | 95 | 44 |
| Mean fraction of HA-stained tumour cells is 32% (SD 29%) | | |

estimated. The fraction of positive tumour cells was primarily analysed semiquantitatively in a continuous scale, but, for statistical calculations, the percentage of tumours cells positive for HA was categorized as seen in Table 2: 0%, <30% and ≥ 30%.

### Statistical analysis

In basic statistical calculations, the SPSS-X program was used in an IBM computer. Frequency distributions were tested by the chi-squared test and Yate's correction was applied when appropriate. The differences between the means of continuous variables were tested by analysis of variance. The univariate survival analysis (log rank analysis, SPSS-X) was based on the life-table method with the statistics of Gehan (Kaplan and Meier, 1958). Multivariate survival analysis (Cox model, Cox, 1972) was carried out with the SPSS-X program package in a stepwise manner, and continuous variables were used as absolute numbers in this analysis. Enter and removal limits were $P \leq 0.05$ and $> 0.10$ respectively.

### RESULTS

Of the 215 patients, 125 were men and 90 women. The mean age of the patients at the time of diagnosis was 66 years (s.d. 12, range 23–89) and the mean follow-up time 10 years (range 4–17). The clinicopathological characteristics of the patients are presented in Table 1.

The expression of HA in the non-neoplastic mucosa in the close vicinity of the tumours was either totally negative or weakly positive. In all cases, the stromal tissue was HA positive (Table 2). Some cases of diffuse-type cancer required careful examination because the neoplastic cells were scattered among the strongly stained stromal tissue, making the evaluation rather difficult. The expression of HA in the tumour parenchyma is summarized in Table 2. In only 15 carcinomas, the parenchyma was totally HA negative, and most of the tumours (79%) showed moderate or strong staining intensity of the neoplastic cells. The different positive staining patterns are illustrated in Figure 1.

The staining intensity of the peritumoral stroma was not associated with any of the examined clinical or histopathological features, except for the fraction of stained cells in the tumour

**Table 3** The association between clinicopathological variables and the HA-staining intensity in the tumour parenchyma of 215 gastric cancer patients

| Variable | – | + | ++ | +++ | Statistics* |
|---|---|---|---|---|---|
| Depth of invasion | | | | | |
| pT1 | 7 | 4 | 12 | 9 | $P = 0.002$ |
| pT2 | 2 | 12 | 15 | 8 | $\chi3 = 30.3$ |
| pT3 | 5 | 13 | 60 | 48 | |
| pT4 | 1 | 1 | 12 | 5 | |
| Nodal status | | | | | |
| pN0 | 0 | 17 | 44 | 26 | $P = 0.61$ (ns) |
| pN1 | 5 | 11 | 39 | 33 | $\chi^2 = 7.2$ |
| pN2 | 1 | 1 | 7 | 7 | |
| Metastasis | | | | | |
| M0 | 15 | 28 | 85 | 53 | $P = 0.066$ (ns) |
| M1 | 0 | 2 | 9 | 15 | $\chi^2 = 11.8$ |
| Laurén classification | | | | | |
| Diffuse | 10 | 7 | 37 | 47 | $P = 0.0002$ |
| Intestinal | 4 | 20 | 43 | 18 | $\chi^2 = 26.6$ |
| Mixed | 0 | 1 | 13 | 6 | |
| Differentiation grade | | | | | |
| Grade I | 4 | 7 | 14 | 3 | $P = 0.010$ |
| Grade II | 1 | 12 | 31 | 10 | $\chi^2 = 16.7$ |
| Grade III | 0 | 2 | 16 | 11 | |
| Tumour location | | | | | |
| Proximal third | 2 | 6 | 22 | 4 | $P = 0.015$ |
| Middle third | 0 | 6 | 12 | 13 | $\chi^2 = 20.4$ |
| Distal third | 11 | 15 | 51 | 34 | |
| More than one-third | 2 | 2 | 14 | 20 | |

*Chi-squared test (Pearson).

($P$<0.0001) and the staining intensity of the tumour parenchyma ($P$< 0.0001)

The staining intensity in the tumour parenchyma was significantly related to pT, as the staining was more intense in deeply penetrating tumours (Table 3). However, no significant association was found between staining intensity and pN or M. Strong staining, as well as negative staining, was more often found among the diffuse-type tumours, whereas the weakly stained tumours were usually of the intestinal type (Table 3). Moderate or strong staining intensity showed a strong association with poorly differentiated tumours of the intestinal type.

The fraction of HA-stained tumour cells was significantly associated with the depth of invasion, the presence of nodal metastases and lymphatic invasion, as well as with differentiation grade as shown in Table 4. HA expression was most frequent in the mixed type of cancer, however without statistical significance. The fraction of HA-positive cells was not significantly related to tumour location, distant metastasis, vascular or perineural invasion or TIL (data not shown).

As shown in Figure 2, the survival of the patients was directly associated with the fraction of HA-stained tumour cells ($P$ = 0.0025). In univariate survival analysis of curatively operated stage I or II patients ($n$ = 110), HA expression did not reach statistical significance. When a multivariate analysis of 195 stage I–IV patients was performed, including several clinical and histological variables (pT, pN, M, size and location of the tumour, vascular, lymphatic and perineural invasion, TIL, Laurén classification, and the fraction of HA-stained cells and staining intensity in the tumour and in the stroma), the independent predictors of survival were pT [ratio of risk (RR) 2.04, 95% confidence interval (CI)

© Cancer Research Campaign 1999 

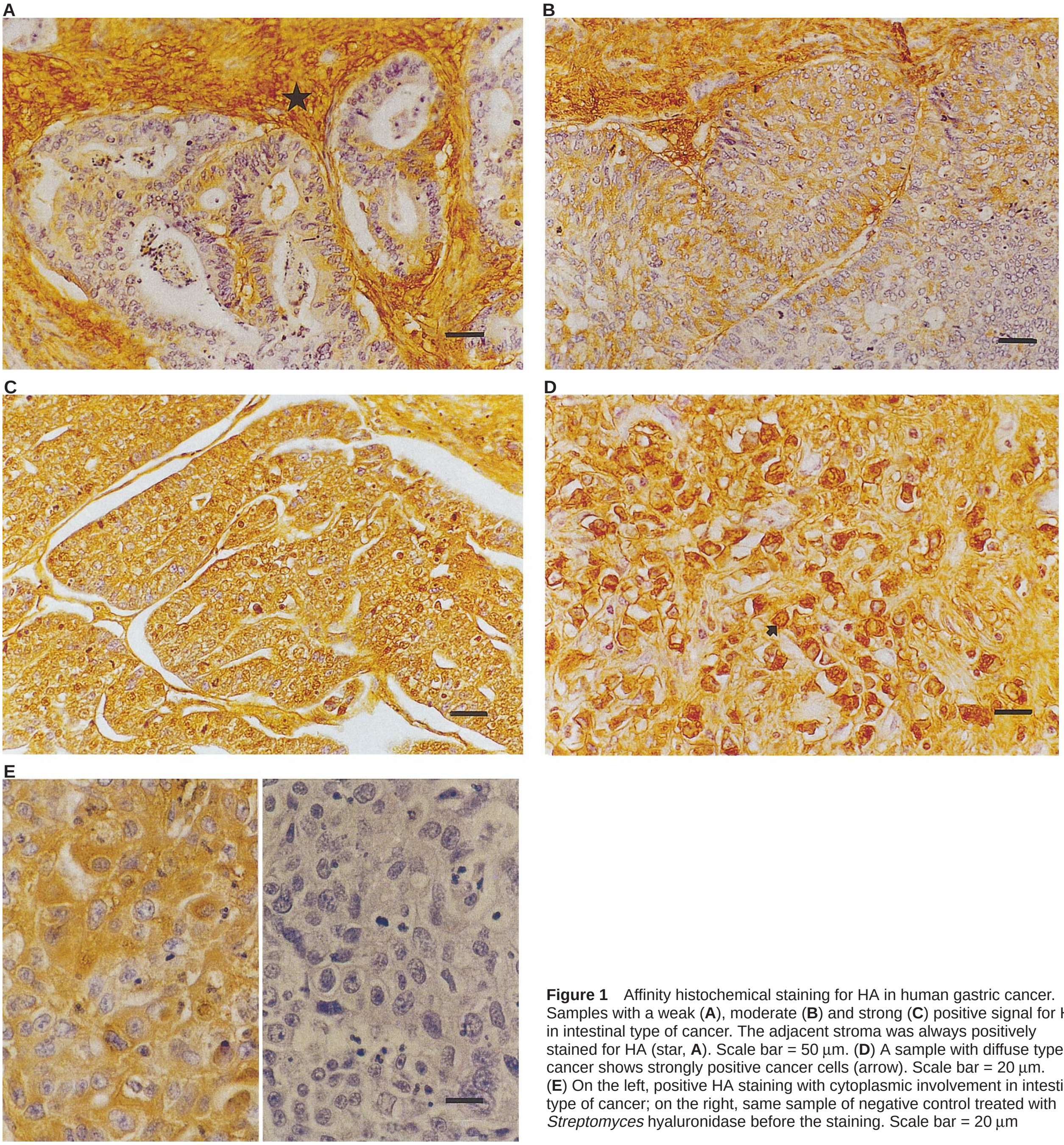

**Figure 1** Affinity histochemical staining for HA in human gastric cancer. Samples with a weak (**A**), moderate (**B**) and strong (**C**) positive signal for HA in intestinal type of cancer. The adjacent stroma was always positively stained for HA (star, **A**). Scale bar = 50 μm. (**D**) A sample with diffuse type of cancer shows strongly positive cancer cells (arrow). Scale bar = 20 μm. (**E**) On the left, positive HA staining with cytoplasmic involvement in intestinal type of cancer; on the right, same sample of negative control treated with *Streptomyces* hyaluronidase before the staining. Scale bar = 20 μm

1.60–2.62], pN (RR 1.75, CI 1.45–2.11), and vascular (RR 2.56, CI 1.49–4.40) and lymphatic invasion (RR 1.38, CI 1.10–1.74), the *P*-value for all being ≤0.005.

### DISCUSSION

The purpose of the present study was to investigate HA expression in different types and stages of gastric adenocarcinoma and to reveal possible associations with malignant behaviour of the tumours. The results indicate that HA is expressed in the neoplastic epithelial cells of most gastric carcinomas (93%), irrespective of the tumour type. The expression of HA was more extensive in advanced cases with deep penetration of the tumour cells in the gastric wall and with nodal metastasis, features known to be associated with poor differentiation grade (Setälä et al, 1996). The frequent abnormal expression of HA in advanced gastric cancer is comparable to the expression of several other molecular abnormalities because the overexpression of growth factor TGF-β and amplification of c-*erb*B-2, K-*sam* and c-*met* are also often encountered in advanced cases (Tahara, 1995). In addition, the expression of the epithelial

 © Cancer Research Campaign 1999

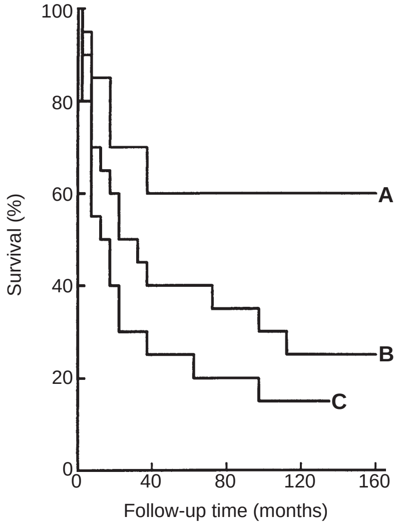

**Figure 2** Survival in stage I–IV gastric cancer patients as related to the fraction of HA-positive tumour cells ($n$ = 215, $P$ = 0.0025, $\chi^2$ = 12.0). (**A**) all tumour cells HA negative ($n$ = 15), (**B**) < 30% of tumour cells HA positive ($n$ = 105), (**C**) 30–100% of tumour cells HA positive ($n$ = 95). (**A** vs **B**: $P$ = 0.096), $\chi^2$ = 2.75. **B** vs **C**: $P$ = 0.0098, $\chi^2$ = 6.67. **A** vs **C**: $P$ = 0.0049, $\chi^2$ = 7.93)

**Table 4** The association between the clinicopathological variables and the mean fraction of HA-positive cancer cells in 215 gastric cancer patients

| Variable | Fraction (%) | Statistics* |
|---|---|---|
| Depth of invasion | | |
| pT1 | 12.2 | $P$ < 0.0001 |
| pT2 | 24.1 | $F$ = 7.4 |
| pT3 | 38.7 | |
| pT4 | 32.1 | |
| Nodal status | | |
| pN0 | 24.7 | $P$ = 0.077 |
| pN1 | 36.8 | $F$ = 4.2 |
| pN2 | 45.3 | |
| Laurén classification | | |
| Diffuse | 32.7 | $P$ = 0.077 (ns) |
| Intestinal | 29.4 | $F$ = 2.6 |
| Mixed | 45.8 | |
| Differentiation grade | | |
| Grade I | 19.6 | $P$ = 0.006 |
| Grade II | 32.7 | $F$ = 5.3 |
| Grade III | 41.6 | |
| Lymphatic invasion | | |
| Negative | 26.2 | $P$ = 0.002 |
| Weakly positive | 36.6 | $F$ = 6.6 |
| Strongly positive | 44.1 | |

*Analysis of variance.

isoform CD44E, containing products of exons v8–v10, or variant CD44, containing v5, has been reported to be significantly associated with deep invasion, nodal metastasis, and lymphatic and vascular invasion (Müller et al, 1997; Rudzki and Jothy, 1997), whereas the expression of CD44v6 is associated with the extent of the disease in the diffuse-type tumours only (Hong et al, 1995).

The current finding that invasion into deeper layers of the gastric wall and to lymphatic structures is frequent in tumours rich in hyaluronan may be explained by the capability of some tumour cells to stimulate HA production in host fibroblasts, causing separation of collagen layers and allowing cell migration and invasion (Knudson et al, 1984). In addition, the cells expressing high levels of HA are easily detached from the primary tumour and may form tumour emboli, enhancing the formation of distant metastases (Catterall et al, 1995). Moreover, the angiogenic effect of HA oligosaccharides (Liu et al, 1996) may significantly enhance cancer growth through tumour neovascularization, an important predictor of disease outcome in gastric cancer (Maeda et al, 1995; Tanigawa et al, 1996).

Significant differences have been reported in the expression of adhesion molecules between the two main types of gastric cancer. The expression of CD44 isoforms containing exons v5 and v6 is more frequent in the intestinal type of cancer (Rudzki and Jothy, 1997), whereas the gene mutations of another adhesion molecule E-cadherin are more prevalent in the diffuse-type cancer (Streit et al, 1996). The above findings seem to be reasonable because the diffuse-type cancer typically invades the gastric wall with single cells or small clusters of cells, whereas the intestinal type is more cohesive in nature (Laurén, 1965). We also hypothesized that we would find more significant differences in the HA expression between the two main types, but failed to do so. The mechanisms behind the different modes of invasion obviously need to be further investigated.

The reports on the relation between HA expression and survival in adenocarcinomas are few so far. However, a recent study by Ropponen et al (1998) found that HA intensity in tumour epithelium was an independent predictor of survival in local colonic adenocarcinoma, concluding that HA may have clinical importance as a prognostic factor in colon cancer. In gastric cancer, some factors of tumour cell adhesion have been proposed to be significant predictors of the disease outcome. The expression of standard CD44 (CD44s) and isoforms containing exons v5 and v9 has been associated with tumour recurrence and high mortality (Mayer et al, 1993; Müller et al, 1997), which is in line with our findings of HA. However, further studies are needed to confirm the impact of abnormal HA expression on survival.

In conclusion, the present study indicates that hyaluronan is expressed in most gastric cancers. Strong HA-expression is significantly related to advanced tumour spread and short survival. From the clinical point of view, the depth of invasion and nodal status remain the most important independent predictors of survival in gastric cancer. However, the present data provide further support to the idea that hyaluronan is one of the key molecules involved in the invasive growth of adenocarcinomas.

### ACKNOWLEDGEMENT

This study has been supported by the EVO funding of Kuopio University Hospital.

### REFERENCES

Catterall JB, Gardner MJ and Turner GA (1995) Hyaluronic acid, cell adhesion and metastasis (review). *Cancer J* **8**: 320–323

Cox DR (1972) Regression models and life tables with discussion. *J Stat Soc B* **34**: 187–192

Hall CL, Yang B, Yang X, Zhang S, Turley M, Samuel S, Lange LA, Wang C, Curpen GD and Savani RC (1995) Overexpression of the hyaluronan

© Cancer Research Campaign 1999

receptor RHAMM is transforming and is also required for H-ras transformation. *Cell* **82**: 19–26
Hong R-L, Lee W-J, Shun C-T, Chu J-S and Chen Y-C (1995) Expression of CD44 and its clinical implication in diffuse-type and intestinal-type gastric adenocarcinomas. *Oncology* **52**: 334–339
Kaplan EL and Meier P (1958) Nonparametric estimation from incomplete observations. *J Am Stat Assoc* **53**: 457–481
Knudson W (1996) Tumor-associated hyaluronan. Providing an extracellular matrix that facilitates invasion (commentary). *Am J Pathol* **148**: 1721–1726
Knudson W, Biswas C and Toole BP (1984) Interactions between human tumor cells and fibroblasts stimulate hyaluronate synthesis. *Proc Natl Acad Sci USA* **81**: 6767–6771
Laurén P (1965) The two histological main types of gastric carcinoma: diffuse and so-called intestinal type carcinoma. An attempt at a histo-clinical classification. *Acta Pathol Microbiol Scand* **64**: 31–49
Liu D, Pearlman E, Diaconu E, Guo K, Mori H, Haqqi T, Markowitz S, Willson J and Sy M-S (1996) Expression of hyaluronidase by tumor cells induces angiogenesis in vivo. *Proc Natl Acad Sci USA* **93**: 7832–7837
Maeda K, Ching Y, Takatsuka S, Ogawa Y, Sawada T, Yamashita Y, Onoda N, Kato Y, Nitta A, Arimoto Y, Kondo Y and Sowa M (1995) Tumor angiogenesis as a predictor of recurrence in gastric carcinoma. *J Clin Oncol* **13**: 477–481
Mayer B, Jauch KW, Günthert U, Figdor CG, Schildberg FW, Funke I and Johnson JP (1993) De-novo expression of CD44 and survival in gastric cancer. *Lancet* **342**: 1019–1022
Müller W, Schneiders A, Heider KH, Meier S, Hommel G and Gabbert HE (1997) Expression and prognostic value of the CD44 splicing variants v5 and v6 in gastric cancer. *J Pathol* **183**: 222–227
Noguchi T, Uchida Y, Oya M, Kubo N and Murakami S (1993) Histochemical study of the extra-cellular stroma in gastric cancer. In *Recent Advances in Management of Digestive Cancers*, Takahashi T (ed.), pp. 263–265. Springer-Verlag: Tokyo
Pauli BU and Knudson W (1988) Tumor invasion: a consequence of destructive and compositional matrix alterations. *Hum Pathol* **19**: 628–639
Ripellino JA, Klinger MM, Margolis RU and Margolis RK (1985) The hyaluronic acid binding region as a specific probe for the localization of hyaluronic acid in tissue sections. *J Histochem Cytochem* **33**: 1060–1066
Rooney P, Kumar S, Ponting J and Wang M (1995) The role of hyaluronan in tumor neovascularization (review). *Int J Cancer* **60**: 632–636
Ropponen K, Tammi M, Parkkinen J, Eskelinen M, Tammi R, Lipponen P, Ågren U, Alhava E and Kosma V-M (1998) Tumor cell associated hyaluronan as an unfavorable prognostic factor in colorectal cancer. *Cancer Res* **58**: 342–347
Rudzki Z and Jothy S (1997) CD44 and the adhesion of neoplastic cells (review). *J Clin Pathol*: *Mol Pathol* **50**: 57–71
Setälä LP, Kosma V-M, Marin S, Lipponen PK, Eskelinen MJ, Syrjänen KJ and Alhava EM (1996) Prognostic factors in gastric cancer: the value of vascular invasion, mitotic rate and lymphoplasmocytic infiltration. *Br J Cancer* **74**: 766–772
Sobue M, Takeuchi J, Tsukidate K, Toida M, Goto K and Nakashima N (1983) Influence of fixed fibroblasts on glycosaminoglycan synthesis of human gastric carcinoma cells in vitro. *Exp Cell Res* **149**: 527–534
Sowa M, Kato Y, Nishimura M, Yoshino H, Kubo T and Umeyama K (1989) Clinico-histochemical study on type 4 carcinoma of the stomach – with special reference to mucopolysaccharides and sialic acid in tumor tissue. *Jpn J Surg* **19**: 153–162
Streit M, Schmidt R, Hilgenfeld RU, Thiel E and Kreuser E-D (1996) Adhesion receptors in malignant transformation and dissemination of gastrointestinal tumors. *J Mol Med* **74**: 253–268
Tahara E (1995) Molecular biology of gastric cancer. *World J Surg* **19**: 484–490
Tammi R, Ågren U, Tuhkanen A-L and Tammi M (1994*a*) Hyaluronan metabolism in skin. *Prog Histochem Cytochem* **29**: 1–81
Tammi R, Rönkkö S, Ågren U and Tammi M (1994*b*) Distribution of hyaluronan in bull reproductive organs. *J Histochem Cytochem* **42**: 1479–1486
Tanigawa N, Amaya H, Matsumura M, Shimomatsuya T, Horiuchi T, Muraoka R and Iki M (1996) Extent of tumor vascularization correlates with prognosis and hematogenous metastasis in gastric carcinoma. *Cancer Res* **56**: 2671–2676
Toole B (1991) Proteoglycans and hyaluronan in morphogenesis and differentiation. In *Cell Biology of the Extracellular Matrix*, ED Hay (ed.), pp. 305–341. Plenum Press: New York
Turley E (1992) Hyaluronan and cell locomotion. *Cancer Metastasis Rev* **11**: 21–30
UICC (1987) *TNM Classification of Malignant Tumours*, Hermanek P and Sobin LH (ed.), pp. 43–45. Springer Verlag: Berlin
Underhill C (1992) CD44: the hyaluronan receptor (commentary). *J Cell Sci* **103**: 293–298
Wang C, Tammi M and Tammi R (1992) Distribution of hyaluronan and its CD44 receptor in the epithelia of human skin appendages. *Histochemistry* **98**: 326–332
Wang C, Tammi M, Guo H and Tammi R (1996) Hyaluronan distribution in the normal epithelium of esophagus, stomach, and colon and their cancers. *Am J Pathol* **148**: 1861–1869

*© Cancer Research Campaign 1999*
